# White-nose syndrome without borders: *Pseudogymnoascus destructans* infection tolerated in Europe and Palearctic Asia but not in North America

**DOI:** 10.1038/srep19829

**Published:** 2016-01-29

**Authors:** Jan Zukal, Hana Bandouchova, Jiri Brichta, Adela Cmokova, Kamil S. Jaron, Miroslav Kolarik, Veronika Kovacova, Alena Kubátová, Alena Nováková, Oleg Orlov, Jiri Pikula, Primož Presetnik, Jurģis Šuba, Alexandra Zahradníková Jr., Natália Martínková

**Affiliations:** 1 Institute of Vertebrate Biology, Academy of Sciences of the Czech Republic, Květná 8, 603 65 Brno, Czech Republic; 2 Department of Botany and Zoology, Masaryk University, Kotlářská 2, 611 37 Brno, Czech Republic; 3 Department of Ecology and Diseases of Game, Fish and Bees, University of Veterinary and Pharmaceutical Sciences Brno, Palackeho 1/3, 612 42 Brno, Czech Republic; 4 Laboratory of Fungal Genetics and Metabolism, Institute of Microbiology, Academy of Sciences of the Czech Republic, Vídeňská 1083, 142 20 Prague 4, Czech Republic; 5 Department of Botany, Faculty of Science, Charles University in Prague, Benátská 2, 128 01 Prague, Czech Republic; 6 Ural State Pedagogical University, Kosmonavtov str. 26, 620017 Yekaterinburg, Russia; 7 Ural State Medical University, Repina str. 3, 620028 Yekaterinburg, Russia; 8 Centre for Cartography of Fauna and Flora, Antoličičeva 1, SI-2204 Miklavž na Dravskem polju, Slovenia; 9 Latvian State Forest Research Institute “Silava”, 111 Rigas str., LV-2169 Salaspils, Latvia; 10 Institute of Molecular Physiology and Genetics, Slovak Academy of Sciences, Vlárska 5, 83334 Bratislava, Slovakia; 11 Institute of Biostatistics and Analysis, Masaryk University, Kamenice 3, 625 00 Brno, Czech Republic

## Abstract

A striking feature of white-nose syndrome, a fungal infection of hibernating bats, is the difference in infection outcome between North America and Europe. Here we show high WNS prevalence both in Europe and on the West Siberian Plain in Asia. Palearctic bat communities tolerate similar fungal loads of *Pseudogymnoascus destructans* infection as their Nearctic counterparts and histopathology indicates equal focal skin tissue invasiveness pathognomonic for WNS lesions. Fungal load positively correlates with disease intensity and it reaches highest values at intermediate latitudes. Prevalence and fungal load dynamics in Palearctic bats remained persistent and high between 2012 and 2014. Dominant haplotypes of five genes are widespread in North America, Europe and Asia, expanding the source region of white-nose syndrome to non-European hibernacula. Our data provides evidence for both endemicity and tolerance to this persistent virulent fungus in the Palearctic, suggesting that host-pathogen interaction equilibrium has been established.

Emerging wildlife infections are a threat to global biodiversity^1^, yet the emergence and transmission of such infectious diseases may also be a function of ecosystem quality and biodiversity^2^. Moreover, anthropogenic disturbance and introductions have been highlighted as contributing considerably to such disease outbreaks^3^,^4^. The majority of emerging wildlife pathogens are of viral origin but fungal infections have also been recognised across a diverse range of taxa, including plants and poikilothermic animals^5^. Relatively few fungal species cause severe diseases in mammals due to their high body temperature and effective immune system^6^,^7^.

Prevalence of pathogens may be high in bat populations^8^,^9^. Despite this, bats have been reported to survive infections that are lethal to other taxa^10^,^11^,^12^. Bat’s capacity to carry pathogens may result from its ability to tolerate damage caused by the agent or the associated immune response^13^,^14^,^15^. Understanding the mechanisms that underlie any trade-off between tolerance and resistance to infectious agents in reservoir hosts is of utmost importance for effective disease control^16^.

Most bats in temperate regions enter seasonal hibernation, at which time they reduce their metabolic rate and decrease body temperature until it approaches the ambient temperature of the hibernaculum, which may alter immune response to pathogens^17^,^18^. During hibernation, bats are vulnerable to infection by the psychrophilic fungus *Pseudogymnoascus destructans* [formerly *Geomyces destructans*]^19^, which emerged as a novel pathogen in eastern North America in 2006^20^. The *P. destructans* fungus is the causative agent of white-nose syndrome (WNS)^20^,^21^,^22^,^23^. Considered an epidemic of major conservation concern in North America, WNS is responsible for an unprecedented decline in bat populations^24^,^25^,^26^. WNS combines some of the worst possible epidemiological characteristics, including a highly virulent pathogen^23^,^27^ with density- and frequency-dependent transmission^28^, an environmental reservoir^19^, long-term persistence in hibernacula^29^,^30^ and susceptibility of multiple hosts^19^,^20^,^31^.

The origin of WNS in North America remains unknown and current research focuses on identifying the infectious agent’s source and identifying whether the agent is an introduced pathogen. Six main lines of evidence support introduction of WNS into North America: 1) Fungal communities associated with bats and their hibernacula in eastern North America comprise a diverse range of *P. destructans* allies^22^,^32^; phylogenetic evaluation, however, indicates that none of these is closely related to *P. destructans*^22^. 2) Previous studies have demonstrated clonal dispersal of a single *P. destructans* genotype among WNS-infected bats in the United States^33^,^34^. 3) Spread of WNS in North America follows a clear invasion front with varying survival rate since first appearance of the disease^24^,^35^,^36^,^37^. 4) Presence of *P. destructans* has been confirmed in many European countries^38^,^39^,^40^,^41^ but with no reports of mass mortality^38^. 5) European *P. destructans* isolates are pathogenic for North American bats^23^. Further, virulent skin infections producing focally severe lesions pathognomonic for WNS have been documented in European bats under natural infection conditions^42^. 6) While only one heterothallic fungal mating type has been recorded in North American *P. destructans* populations, two types have been found coexisting in European hibernacula. Effective recombination during sexual reproduction results in genetic variability and may be linked with virulence^43^. These indirect sources of evidence tend to support the introduced pathogen hypothesis, suggesting WNS may have originated outside of North America^22^,^23^,^43^,^44^. Comparative studies between North America and Europe have been proposed to explain the origin and differential manifestation of the fungal infection^4^,^40^,^42^,^45^.

Although some authors speculate on a non-European origin^4^,^25^, Europe is believed to be the likely source of WNS^44^. Supporting evidence for long exposure to the WNS fungus based on old European photographs, which appear to show a white cutaneous fungal infection^38^,^40^, lacks specificity for WNS. Another dermatophyte, *Trichophyton redellii*, produces a similar gross appearance in hibernating bats^46^ and the photographs might represent either. On the contrary, presence of WNS in multiple and diverse hosts^31^ indicates that the WNS fungus could persist across their ranges in the Palearctic.

To date, WNS diagnostic skin lesions have only been reported from the Czech Republic, Europe, with almost half of all species being WNS positive and prevalence based on histopathology reaching 55% in bats emerging from hibernacula in spring^31^,^42^. Occurrence of WNS in distantly related bat species with diverse ecologies, however, suggests the pathogen is a generalist and that all bats hibernating within the distribution range of *P. destructans* may be at risk of infection^31^. If we assume that *P. destructans* range expansion and its concurrent WNS epidemic occurred unnoticed in Europe in the past, we expect that WNS should be found at any site in Europe with conditions favourable for the pathogen and could extend to non-European hibernacula of the Palearctic region. We tested the following four hypotheses. First, *P. destructans* is present in European and non-European Palearctic hibernacula and, if the fungus is present in a hibernaculum, bats would test positive for WNS under histopathology. Second, an endemic steady-state of *P. destructans* infection in the Palearctic would be reflected in a persistent high prevalence among bats with no associated population declines. Third, given that the differences in population size changes in WNS-positive regions^24^,^38^ are explained as WNS resistance in Europe, Palearctic bats will display a lower pathogen load and smaller size of cupping erosions diagnostic for WNS than Nearctic species. Fourth, in regions with endemic pathogen occurrence, *P. destructans* load will be influenced by geographic location, sampling date and bat community structure.

## Results

### WNS in the Palearctic

**Figure 1:**
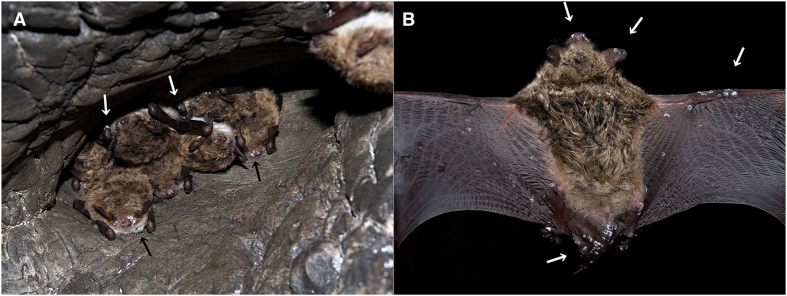
Fungal growth on hibernating bats from Russia. (**a**) A hibernating cluster of pond bats *Myotis dasycneme* in a cave near Yekaterinburg, Russia, in May 2014. Black and white arrows indicate fungal growth on the muzzle and forearm, respectively. (**b**) A pond bat from the same hibernaculum showing visible fungal growths on the uropatagium, pelvic limb toes, plagio- and pro-patagium and the ears and muzzle (white arrows). Photo: Jiri Pikula.

**Figure 2:**
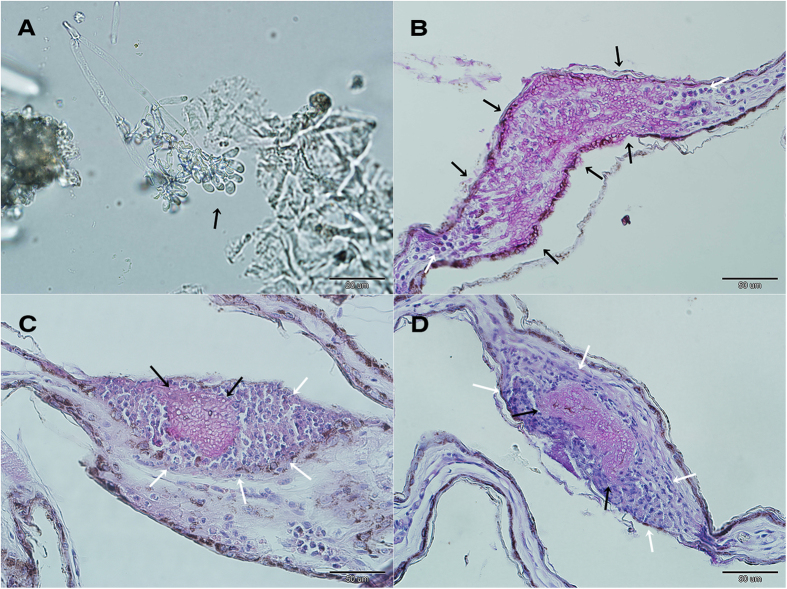
White-nose syndrome on a pond bat *Myotis dasycneme* near Yekaterinburg, Russia, in May 2014. (**A**) Microscopic identification of the characteristic curved conidia of *Pseudogymnoascus destructans* (black arrow). (**B**) Invasive fungal growth penetrating the full-thickness of the wing membrane (black arrows), with several inflammatory cells (neutrophils) situated at both margins of the lesion (white arrows). (**C**) Packed fungal hyphae of cupping erosions (black arrows) sequestered by neutrophils (white arrows). (**D**) Histopathological finding from a WNS-positive Nearctic *Myotis lucifugus*, identical to that found in Palearctic Asia. Skin sections stained with periodic acid-Schiff stain.

We sampled and examined bats from sites in geographically distant regions (the Czech Republic, Slovenia, Latvia and Russia; *n* = 481) to assess occurrence of WNS in the Palearctic (Table 1, Supplementary Table S1). We found WNS-positive bats from multiple species at all sites, including the West Siberian Plain in Russia. At each site, all of the following criteria categorised the site as WNS positive: suspected fungal growth (Fig. 1), characteristic curved conidia of *P. destructans* observed on an adhesive tape imprint (Fig. 2A), *P. destructans* confirmed by DNA sequencing and qPCR using a species-specific probe, and definitive diagnosis confirmed through histopathology of biopsy punches from wing membrane lesions targeted by UV trans-illumination (Fig. 2B).

Histopathological findings matching WNS diagnostic criteria were present in 100 bats of 13 different species (Table 1). Two species were newly identified as positive for WNS, *Miniopterus schreibersii* and *Rhinolophus euryale*. Species-specific prevalence, using qPCR to detect *P. destructans* and UV trans-illumination to detect lesions indicative of WNS, ranged from 64 to 100% (overall prevalence = 83.1%) and 14 to 100% (overall prevalence = 51.9%), respectively (Table 1).

**Figure 3:**
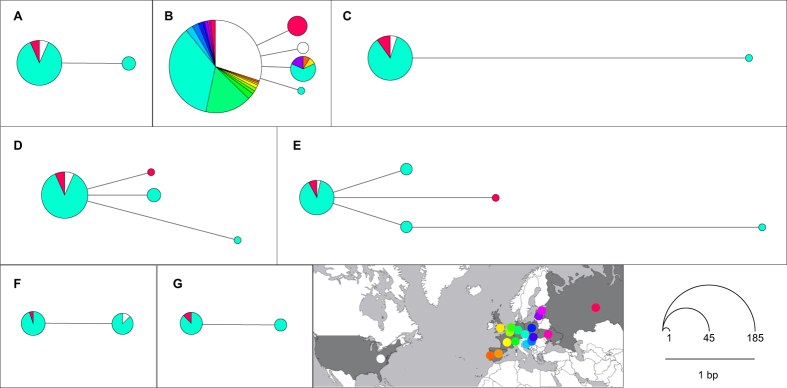
Median-joining networks for *Pseudogymnoascus destructans* DNA sequences. Node centroid distances correspond to the number of substitutions between haplotypes and the node size to the number of isolates sharing the haplotype. Isolate origin is signified through different colours based on the inset map. The map was modified from http://www.amcharts.com/visited_countries/ (last accessed on 9 October, 2015). (**A**) *TUB2* (*n* = 46), (**B**) *ITS* (*n* = 203), (**C**) *CAM* (*n* = 47), (**D**) *TEF1α* (*n* = 51), (**E**) sequences concatenated with *TUB2*, *ITS*, *CAM* and *TEF1α* (*n* = 34); haplotypes pooled according to the available sequence and gaps treated as missing data, (**F**) *MAT1-1-1* (*n* = 27), (**G**) *MAT1-2-1* (*n* = 19).

In order to evaluate *P. destructans* genetic variability, we sequenced six loci resulting in 254 new sequences. Two alleles were found in *TUB2*, *MAT1-1-1*, *MAT1-2-1* and *CAM* loci, four alleles in *TEF1α* and three in *ITS* (Fig. 3). The most frequent alleles from all loci were found in both European and Asian *P. destructans* isolates. In *ITS* and *TEF1α* from Asia, we sampled isolates with one substitution difference from the most frequent allele. The dominant haplotype from the concatenated sequence of four genes (*TUB2*, *CAM, TEF1α*, *ITS*) is widespread in North America, Europe and Asia (Fig. 3E). Three divergent concatenated haplotypes were found in the Czech Republic and one local to Asian Russia. The *P. destructans* mating type proportion was 27 (*MAT1-1-1*) to 19 (*MAT1-2-1*) (χ^2^ = 1.391, *P* = 0.238).

### Quantitative comparison of WNS on bats

The fungal load on qPCR-positive bats ranged from 0.21 pg to 3.41 μg across the surface of the left wing (Supplementary Fig. S1, see Table 1 for sample sizes). This range included fungal loads reported from Nearctic bat species (Supplementary Fig. S1). Log-transformed fungal load was not lower on Palearctic bats than on Nearctic bats (Wilcoxon *W* = 68030, *P* = 1, *n* = 413 and 247, respectively), even assuming that fungal load on Nearctic bats could vary up to ten-times due to the difference in sample collection method between continents (Wilcoxon *W* = 50910, *P* = 0.484).

**Figure 4:**
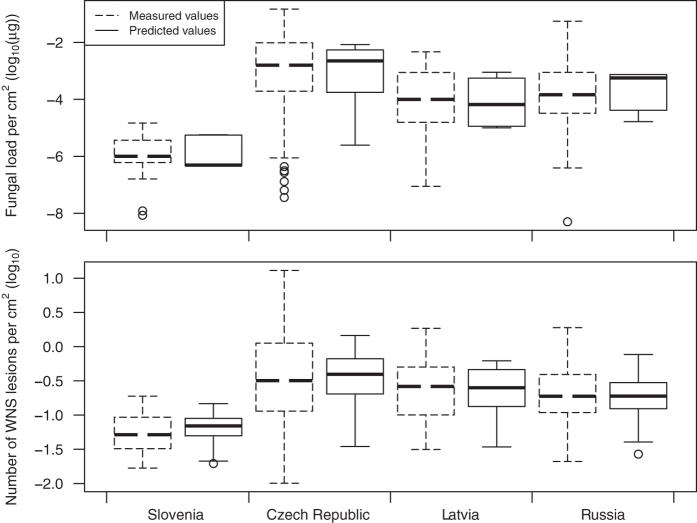
Fungal load and number of WNS lesions in Europe (Czech Republic, Latvia, Slovenia) and in Asia (Russia). Fungal load is quantified as *P. destructans*-specific DNA per cm^2^ of wing area (established through quantitative PCR) and number of WNS lesions is counted on the same wing (using UV light trans-illumination).

**Figure 5:**
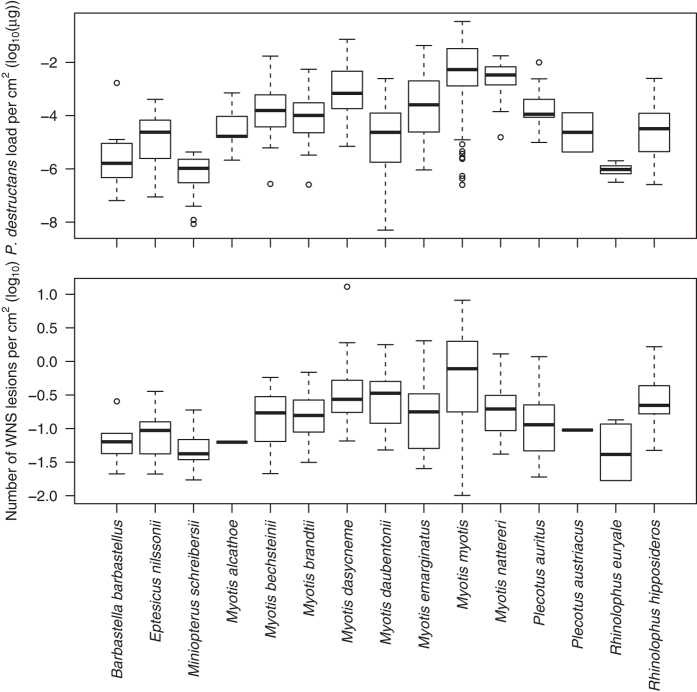
Fungal load and number of WNS lesions on bats from Europe (Czech Republic, Latvia, Slovenia) and Asia (Russia). Fungal load is quantified as *P. destructans*-specific DNA per cm^2^ of wing area (established through quantitative PCR) and number of WNS lesions is counted on the same wing (using UV light trans-illumination).

In order to account for body size differences between Palearctic bat species, we used log-transformed fungal load per cm^2^ of wing area (henceforth referred to as fungal load, unless specified otherwise). Fungal load differed significantly between regions (Kruskal-Wallis *χ*^2^ = 94.2, *P* < 0.001; Fig. 4) and species (Kruskal-Wallis *χ*^2^ = 221.2, *P* < 0.001; Fig. 5), with highest values recorded in the Czech Republic and two euryvalent bat species, *Myotis myotis* and *Myotis nattereri*, respectively. In frequently captured *M. myotis*, fungal load did not change between 2012 and 2014 (ANOVA: *F*_2,146_ = 1.209, *P* = 0.301). Prevalence dynamics of nine species captured in multiple years was statistically equal between years (*χ*^2^ test: *P* > 0.05; Czech Republic). The sampled regions contained multiple species with varying fungal loads (Fig. 5), and some species were present in multiple regions (Table 1). The observed difference of fungal load can be caused by a different set of species or different environmental conditions in sampled regions. Therefore, we separated the effect of the two variables on fungal load by correcting for the random effect of species and region. This had no impact on significance of the comparison between regions or species. The phylogenetic signal of mean species fungal load was significant (Blomberg’s *K* = 0.892, *P* = 0.002), meaning that closely related taxa had more similar mean fungal loads in the Palearctic than expected by chance.

**Figure 6:**
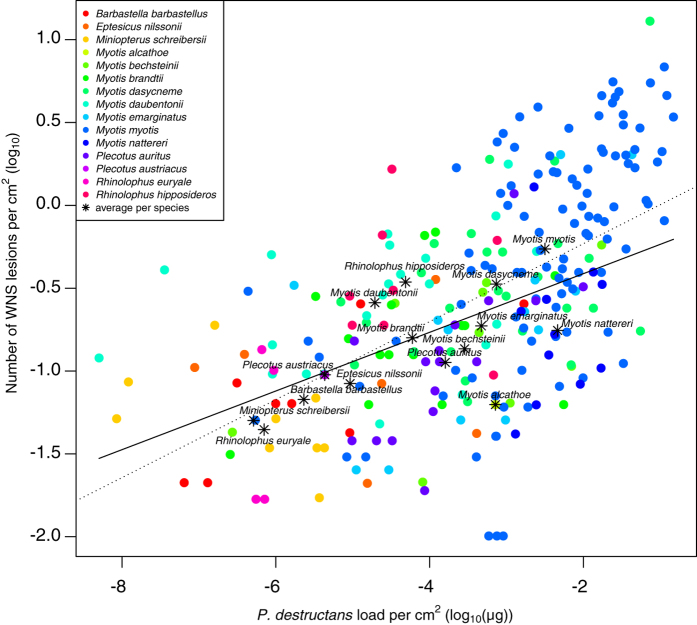
Phylogenetic generalised least-squares for number of WNS lesions, dependent on fungal load and accounting for within-species variation and relatedness. The dotted line represents the linear regression without phylogenetic correction.

Fungal load was significantly correlated with the number of WNS lesions (Spearman’s rank correlation: *r* = 0.61, *P* < 0.001). Phylogenetic generalised least-squares, which accounts for intra-specific variability and species phylogenetic relationships, indicated that the number of WNS lesions increased with increasing fungal load across bat diversity in this study (intercept = –0.058, slope = 0.177; Fig. 6). No significant phylogenetic signal was detected in the mean number of WNS lesions by species (Blomberg’s *K* = 0.511, *P* = 0.073).

**Figure 7:**
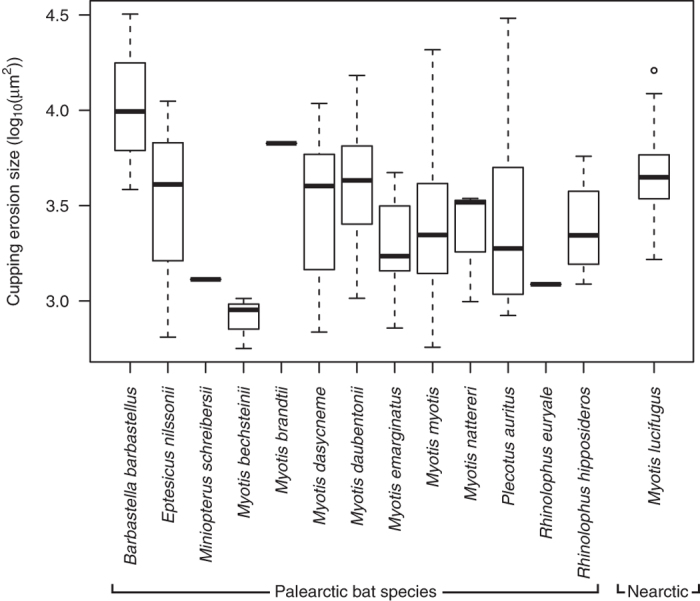
Variation in focal tissue invasiveness among hibernating bat species infected by *Pseudogymnoascus destructans*. A series of 80 periodic acid–Schiff stained sections from each bat’s wing membrane biopsy were used to determine mean maximum size of WNS-diagnostic cupping erosions (log_10_(μm^2^)). See Table 1 for sample sizes.

Microscopic WNS cupping erosion width ranged between 28.6 and 397.2 μm (median = 86.34 μm, *n* = 105) and depth between 11.3 and 91.8 μm (median = 31.29 μm), this also being the WNS-lesion size range detectable photographically as individual spots using UV trans-illumination. Size of WNS lesion (log-transformed) did not differ significantly between species (Kruskal-Wallis *χ*^2^ = 20.7, *P* = 0.079, *n* = 14 species across the Holarctic; Fig. 7, see Table 1 for sample sizes). Phylogenetic signal for mean species-specific WNS lesion size was also not significant (Blomberg’s *K* = 0.448, *P* = 0.177) and there was no significant difference observed between Palearctic and Nearctic bats (phylogenetic ANOVA: *F* = 0.43, *P* = 0.292).

**Figure 8:**
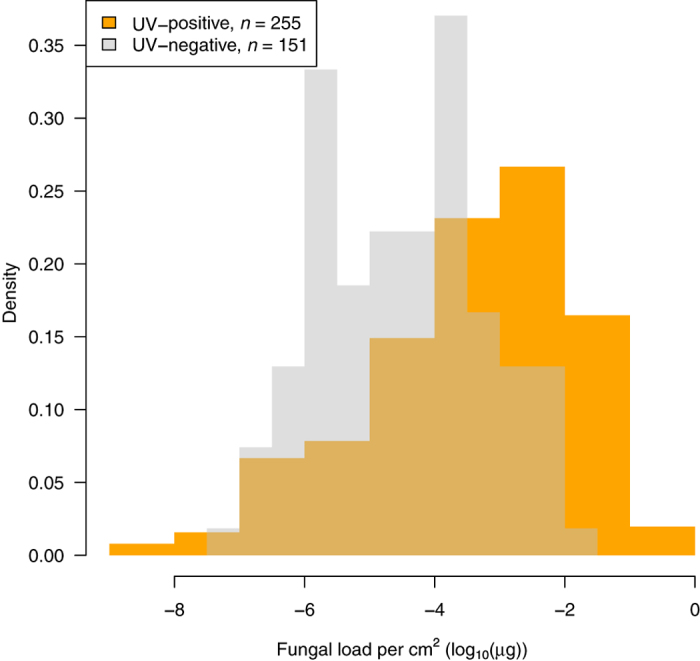
Frequency of *Pseudogymnoascus destructans* fungal load per cm^2^ of wing area. Bats were identified as positive (grey; *n* = 255) and negative (orange; *n* = 151) using UV trans-illumination.

The number of WNS lesions in animals positive over UV ranged from 1 to 805 (median = 13) and differed significantly between Palearctic regions (log-transformed per cm^2^; Kruskal-Wallis *χ*^2^ = 33.6, *P* < 0.001; Fig. 4) and species (Kruskal-Wallis *χ*^2^ = 76.7, *P* < 0.001; Fig. 5; correction did not influence significance). The fungal load from UV-negative individuals (median = 3.78 × 10^−5^ μg) overlapped that from UV-positive individuals (median = 7.46 × 10^−4^ μg; Fig. 8).

Comparison of AIC values indicated that the best fitting model for fungal load across regions included geographic coordinates (95% confidence interval of parameter estimates: longitude: –0.07–(–0.03), latitude: 0.16–0.36), sampling date (0.002–0.02) and structure of common bat species community found in the respective region (–3.86−(−2.24)) (Supplementary Table S2). On the other hand, disease intensity, quantified as number of WNS lesions, was best modelled using fungal load (0.18–0.28) and sampling date (–0.007–0.0003), though addition or removal of other variables did not change the relationship between WNS-lesion number and fungal load significantly (0.18–0.27). In both cases, species was treated as a random effect. Values predicted from the optimal model for both fungal load and number of WNS lesions were highest in the Czech Republic (Fig. 4). Lowest values were predicted in Slovenia and intermediate values in Latvia and Russia.

## Discussion

Here we show WNS infection in Palearctic bat communities within and beyond the borders of Europe, greatly extending the distribution range of *P. destructans* and confirming its generalist nature. Furthermore, we confirm WNS skin lesions in two more bat species, belonging to families Miniopteridae and Rhinolophidae.

Histopathology of skin sections is presently considered the ‘gold standard’ for diagnosing WNS^47^. The microscopically identified WNS in bat species sampled in Russia was consistent with WNS histopathological criteria^42^,^47^. These included characteristic cup-shaped epidermal erosions, *P. destructans*-infected hair follicles, associated sebaceous glands and regional connective tissues, and invasive fungal growth throughout the wing-membrane thickness (Fig. 2). Our results demonstrate that *P. destructans* is virulent for Palearctic bats under natural infectious conditions, as previously shown in the Czech Republic^31^,^42^. Local invasiveness and WNS-lesion severity in wing membranes of bats collected in the Palearctic was comparable with that of Nearctic bats (Figs 2 and 8), with diagnostic features ranging from cupping erosions to full-thickness fungal invasion found on all continents. In our experience, detection of WNS-positive bats was greatly enhanced by the use of a UV lamp combined with non-lethal punch biopsies of suspected skin lesions^48^. The effectiveness of this method enabled us to identify seven species as WNS-positive in multiple regions, i.e. *Myotis brandtii*, *Myotis dasycneme*, *Myotis daubentonii*, *Myotis emarginatus*, *M. myotis*, *Eptesicus nilssonii* and *Plecotus auritus* (Table 1). As predicted^31^, *M. schreibersii* and *R. euryale* were confirmed positive for skin lesions. These are thermophilic species that only hibernate in the northern part of their ranges. *Miniopterus schreibersii*, a long-distance migrant, forms large mixed colonies with other cave-dwelling bat species^49^ and such characteristics make the species another effective candidate for pathogen dispersal.

Theoretically, WNS spread is limited by conditions prevalent in underground hibernacula being favourable to *P. destructans* and presence of susceptible hibernating bats^50^. *Pseudogymnoascus destructans*, being a generalist pathogen, could infect any bat species hibernating under the right microclimatic conditions and ecological and evolutionary differences in the hibernating bats would not pose a barrier^31^. Distribution ranges of multiple WNS-positive bat species (*E. nilssonii*, *M. dasycneme*, *M. daubentonii*, *M. brandtii*, *P. auritus*) extend across Palearctic Asia and overlap with their sister species or ecological counterparts (e.g. *Myotis petax*, *Myotis sibiricus*). As bats can form multi-species clusters at hibernation sites, there is a good chance that *P. destructans* could switch host species to presently WNS-negative taxa (e.g. *Myotis ikonnikovi*, *M. petax*, *M. sibiricus*). This suggests that WNS may be present throughout the Palearctic where suitable environmental conditions occur and in all bat species that utilise such sites. In fact, a paper published ahead of print during review of this article corroborates our conclusions with one *M. petax* found positive for WNS in North China^51^.

Given the tragic impact of WNS on bat biodiversity in North America^24^, it is important to identify the source of possible introduction^4^. However, *P. destructans* exhibits notoriously low genetic diversity in both the clonal North American populations^33^,^34^,^43^ and the sexually reproducing populations of Europe^44^, effectively thwarting efforts to pinpoint the source region. Our study further exacerbates the enigma of where the North American *P. destructans* strain originates as the North American haplotype was found in all non-mating gene types in Palearctic Asia, thereby expanding the putative source region (Fig. 3). Additionally, the search for a source region should shift to markers with a faster mutation rate and better resolution for *P. destructans* phylogeography, as the low genetic variability in markers routinely used in fungal population studies indicates a relatively recent origin or expansion of the Palearctic population.

Theory suggests that if a disease is detectable at high prevalence it is probably mild and unlikely to be a major problem^52^. Two methods were used to establish prevalence in this study: qPCR, used to quantify the pathogen on wing skin^53^, and UV trans-illumination of the wing membrane, which enables detection of fluorescing lesions associated with WNS skin infection^48^. Both methods provided comparable results of high prevalence, and, together with stable or increasing host population sizes^54^, our data strongly suggest endemicity of *P. destructans* within Palearctic bat populations. Persistent high prevalence and pathogen load with absence of population declines in the Palearctic are in sharp contrast to the situation in the Nearctic. In Midwestern United States, high prevalence of *P. destructans* infection was followed by a decrease in number of hibernating bats^55^. Some form of host-pathogen equilibrium, analogous to the amphibian chytrid fungus observed in post-decline frog communities^56^, may already have occurred in the Palearctic but not in the Nearctic.

Pathogen load indicates both exposure to the infectious agent and suitability of the host for pathogen replication^57^. *Pseudogymnoascus destructans* fungal load can be evaluated using swab samples from wing membranes^53^. Although swabs do not sample fungus invasion deep in the wing tissue, our results show that fungal load sampled in this way is positively correlated with the number of WNS lesions (Fig. 6). Hence, fungal load from swabs may be assumed to approximate the total load associated with the skin infection. Fungal loads determined across the Palearctic were similar to those observed in Nearctic species (Supplementary Fig. S1). The swabbing technique used in the North American studies^19^,^58^,^59^, however, does not allow standardisation of *P. destructans* load per cm^2^ and thus detailed statistical comparisons cannot be made. Nevertheless, fungal loads in Nearctic bats are similar to the observed fungal load of Palearctic bats (Supplementary Fig. S1), even if the former were underestimated ten-fold with the different swabbing technique.

In the Palearctic study area, fungal load differed regionally (Fig. 4) and between species (Fig. 5) with no reported mass decline in bat populations attributable to WNS^38^,^54^. Fungal load increased with increasing northing and westing, sampling day (load increasing later in the year) and in regions where phylogenetically closely-related hibernating bats predominated (Fig. 4). The driving factor that best modelled the number of WNS lesions observed using UV trans-illumination in the Palearctic was the fungal load. Increasing fungal load positively correlated with disease intensity across species diversity indicates that hyphae are more likely to invade deep tissues and cause WNS lesions with heavy fungal growth on bat wings (Fig. 5). However, with overlap of fungal loads in UV-negative and UV-positive bats, no clear fungal load threshold defines the pathogen pressure where development of WNS lesions starts.

With persistent and similar fungal load (Supplementary Fig. S1) and cupping erosion size (Fig. 7) on bats from the Palearctic and the Nearctic, the difference in population size response is striking. While population sizes dropped dramatically under the WNS epidemic in the Nearctic^24^, population size changes remained within normal inter-annual fluctuation levels in the Palearctic^38^,^54^,^60^. Our data suggest that, once the hibernacula are contaminated by the fungus, Palearctic bats are exposed to high pathogen pressure and the infection is continuously present to a high level, i.e. the so-called hyperendemic condition. The difference in infection outcome is therefore a function of adaptation to pathogen pressure.

While reduction of pathogen load is a function of host resistance, the ability to limit the harm caused by a given load is a result of tolerance^61^,^62^. These two alternative and complementary forms of defence may have profound effects on the epidemiology of infectious diseases and on host-pathogen coevolution^62^. Resistance protects the host at the expense of the pathogen and tolerance saves the host from harm without direct negative effects on the infectious agent. Evolution of resistance, therefore, should also reduce the prevalence of the pathogen in host populations. On the other hand, tolerance is expected to have a neutral, or even positive, effect on prevalence of the pathogen. Lack of resistance to WNS infection in hibernating European bats has been demonstrated in 13 species^31^,^42^ that exhibit stable or increasing population sizes^54^. Yet, prevalence of *P. destructans*-positive bats reached 100% in the Palearctic. In light of the above arguments, it would appear that mechanisms promoting tolerance to *P. destructans* infection are in operation in the area studied. Palearctic bat species tolerate comparable fungal loads and WNS-lesion size to their Nearctic counterparts suffering population declines. We hypothesise that the balance between tolerance and resistance mechanisms changes with transition of bats from hibernation to euthermia.

Our results provide evidence of *P. destructans* and pathognomonic WNS skin lesions in hibernating bats sampled from the West Siberian Plain of Russian Asia and indicate endemicity of this virulent fungus in the Palearctic region. Data suggesting bat tolerance imply lowered risk following establishment of equilibrium in the host-pathogen interaction. The extensive spatial distribution of the agent may pose a threat, however, representing a continued source of introduction to other regions with naïve bats not yet exposed to the pathogen. Classical models employing the disease triangle concept suggest that the epidemiological outcome of an infection depends on determinants of the pathogen, host(s) and the environment. Alterations in any of these determinants may trigger shifts in the complex host-pathogen system, as seen here by infection tolerance in hibernating Palearctic bats.

## Methods

### Material collection

Between 2012 and 2014, we sampled 481 bats (15 species) at 20 sites in Slovenia, the Czech Republic, Latvia and Russia (West Siberian Plain, Asia; Table 1, Fig. 3). Samples were taken as late in the hibernation or as early in the post-hibernation season as possible (February-May) to minimise the impact of disturbance to specific bat species. Following capture, the wings, ears and muzzle were swabbed with a nylon (FLOQ Swabs, Copan Flock Technologies srl, Brescia, Italy) or cotton swab (Plain swab sterile plastic applicator, Copan) in a standardised manner in order to collect fungal biomass from the whole skin area for fungal detection (dorsal side of left wing only for qPCR). The bats were photographed over a 368 nm wavelength UV lamp and wing punch biopsies of suspect tissue collected in 10% formalin for histopathological examination. All bats were then released at the site.

WNS was diagnosed according to current standards^47^, i.e. *P. destructans* presence was confirmed with qPCR^53^ and selected orange-yellow spots observed over UV were sampled. A series of 80 periodic acid-Schiff stained sections embedded in paraffin were obtained from each wing membrane biopsy. These were then observed under an Olympus BX51 light microscope (Olympus Corporation, Tokyo, Japan). Fungal cell walls were stained magenta under the periodic acid-Schiff stain, which allowed measurement of cupping erosions packed with hyphae. Using cellSense Software tools (Olympus Soft Imaging, GmbH, Münster, Germany), we measured total area (size) of cupping erosions. Trans-illuminated photographs of the left wing membrane stretched over a UV lamp were used to manually enumerate yellow-orange fluorescing pinpoints indicative of WNS lesions^48^, using the individual object counting tool of ImageJ^63^.

Bats were considered WNS-positive if qPCR confirmed *P. destructans* infection, wings exhibited characteristic UV fluorescence^48^ and cupping erosions packed with fungal hyphae or a full thickness fungal invasion of the wing membrane were observed under histopathology^47^. Additional swabs taken from the wings and muzzle after sampling for qPCR were used for cultivation on Sabouraud dextrose agar plates and isolation of the fungus in pure cultures^38^. Representative isolates were deposited at the Culture Collection of Fungi, Charles University in Prague, Czech Republic.

Field work and sampling in the Czech Republic was performed in accordance with Czech Law No. 114/1992 on Nature and Landscape Protection, based on permits 01662/MK/2012S/00775/MK/2012, 866/JS/2012 and 00356/KK/2008/AOPK issued by the Agency for Nature Conservation and Landscape Protection of the Czech Republic. Experimental procedures were approved by the Ethical Committee of the Academy of Sciences of the Czech Republic (No. 169/2011). Sampling in Latvia was approved by the Nature Conservation Agency (No. 3.15/146/2014-N), in Slovenia by the Ministry of Environment and Spatial Planning of the Slovenian Republic, Slovenian Environment Agency (No. 35601-35/2010-6) and in Russia by the Institute of Plant and Animal Ecology, Ural Division of the Russian Academy of Sciences (No. 16353–2115/325). The authors were authorised to handle free-living bats according to the Czech Certificate of Competency (No. CZ01341; §17, Act No. 246/1992 Coll.) and a permit approved by the Latvian Nature Conservation Agency (No. 05/2014).

### DNA isolation

Total DNA was isolated from swabs using the QIAamp DNA Mini Kit (Qiagen, Halden, Germany). Swabs were placed in tissue lysis buffer with proteinase K and incubated at 56 °C for two hours. Lysis buffer was added and the samples incubated for a further 10 min. After this step, we followed the Qiagen Buccal Swab Spin Protocol according to the manufacturer’s recommendations.

### Quantitative PCR

We performed quantitative PCR^53^ (qPCR) using TaqMan® Universal Master Mix II with UNG (Life Technologies, Foster City, CA, USA). To optimise the PCR reaction, we added bovine serum albumin and Platinum® Taq DNA Polymerase (Life Technologies) in final concentrations of 0.05 mg/μl and 0.025 U/μl, respectively. We used both forward and reverse primers (0.3 μM each) and species-specific and genus-specific fluorescently-labelled custom probes were used for quantification of the PCR product. The reaction mix was prepared on ice and three replicates were prepared for each DNA sample. We performed real-time PCR reaction on a LightCycler 480 PCR platform (Life Technologies) with initial inactivation at 50 °C for 2 min and a hot start at 96 °C for 10 min. Nine cycles with a denaturation step at 95 °C for 15 sec and annealing at 62 °C for 1 min were followed by 43 identical cycles with quantification detection. qPCR was finalised by dissociation at 95-60-95 °C for 15 sec each and cooling to 40 °C for 10 min. DNA isolated from CCF3937 culture^38^ and water were used as positive and negative controls and as concentration references for each run.

### Fungal load on Palearctic bats

We calculated a DNA concentration calibration curve using a CCF3937 dilution series. Exact DNA concentrations (ng μl^−1^) in the dilution series were determined using Qubit HS fluorometry via the manufacturer’s protocol. Effectivity of qPCR was 1.96. We used this dilution series to estimate the relationship between fungal load and qPCR cycle, calculating *P. destructans* DNA concentration in the sample using custom scripts in R^64^ with the equation log(*q*_*PdDNA*_) = 3.194 – 0.287 *Cp* (*R*^2^ = 0.9719), where *q* is the DNA concentration and *Cp* the cycle. Each result was converted to fungal load based on the positive control and overall elution of DNA.

### Fungal load on Nearctic bats

We downloaded previously published *P. destructans* loads from natural infections^19^,^58^ or recalculated loads from mean *Cp* values according to the authors’ equation^59^. In one case, we plotted means as individual data points as the authors^19^ included species means per site (plus standard error) but fungal loads per individual were not published. The sample sizes for Nearctic bat species sampled within the dates used in this study were: *Corynorhinus rafinesquii* (*n* = 2), *Eptesicus fuscus* (*n* = 6), *Lasiurus borealis* (*n* = 2), *Myotis grisescens* (*n* = 11), *M. leibii* (*n* = 1), *M. lucifugus* (*n* = 36), *M. septentrionalis* (*n* = 4), *M. sodalis* (*n* = 13) and *Perimyotis fuscus* (*n* = 172). None of the North American studies specified sampled area on the bat with sufficient precision to enable standardisation to cm^2^ or universal statistical comparison.

### Molecular genetic variability of *P.* destructans

To assess genetic variability, we used a set of isolates collected between 2009 and 2014 in the Czech Republic^38^,^65^ and isolates from this study. The isolates were characterised using *ITS* and five other nuclear gene sequences using previously published primers (Supplementary Table S3). Chromatograms were assembled to DNA sequences in Geneious 6 (Biomatters, Auckland, New Zealand) and submitted to the European Nucleotide Archive (LN871244-LN871428). These were checked with blast and *P. destructans* was confirmed at all sites with a sequence identity to previously sequenced strains ≥99% in all markers. We then downloaded previously published *P. destructans* sequences of the respective genes from GenBank. Sequence haplotypes were identified based on available nucleotide residues, with gaps and unresolved bases treated as missing data. We estimated relationships between haplotypes using median-joining networks^66^.

### Individual-based linear mixed models

We modelled intensity of WNS infection using fungal load on the dorsal side of the left wing and the number of WNS lesions on the same wing visualised over UV in R^64^. UV-detectable lesions correspond with cupping erosions diagnostic for WNS in Europe and North America, thereby reflecting disease intensity^47^,^48^. Our previous experience in the Czech Republic has shown that wing damage tends to be localised and that lesions do not usually merge^31^, allowing them to be counted. The yellow-orange fluorescent spots were counted by a researcher with no knowledge of the qPCR and histopathology results for the samples. We scaled variables to 1 cm^2^ of wing area^67^ and log-transformed them in order to account for body-size differences between species. We included latitude and longitude, sampling day (from the beginning of the calendar year) and scores for the first two principal components (characterising dominant hibernating bat community in each region; Supplementary Fig. S2) as fixed-effect variables and species as a random effect. Microclimate at a specific bat roost might influence fungal load and disease intensity on an individual through a compound effect of optimizing growth conditions for the pathogen and hibernation conditions for the host. We used geographic coordinates as a proxy for possible general site microclimate (mean annual temperature, elevation, humidity) under the assumption that fungal load and number of WNS lesions would increase at higher latitudes and with longitudinal shift from oceanic to more continental climate. Similarly, we expected day of sampling to be reflected in the dependent variables as increased time for fungus propagation might increase its loading. As such, geographic location, combined with sampling date, can be understood as a proxy for cave microclimate, which would influence growth of the WNS fungus^50^,^68^.

Both relatedness and species assemblage differences at hibernacula in a given region could skew regional fungal load and disease intensity in favour of those areas where severely infested species co-occur. We estimated bat communities present at hibernacula from local surveys. Relatedness of species recorded was assessed using mean phylogenetic distance and mean nearest taxon distance (both with weighted abundance) and UniFrac metric measuring the percentage of phylogeny shared by a given community. We used these metrics for principal components analysis. We used backward stepwise variable selection to develop the model, which was assessed using the Akaike Information Criterion (AIC).

### Species-level phylogenetically-informed model

Previously published phylogenetic trees for European bats^31^ based on multilocus sequence data were rescaled as an ultrametric tree using penalised likelihood^69^,^70^ with λ and root height = 1. We used R^70,71^ to calculate phylogenetic signal in species means and phylogenetic ANOVA^71^,^72^. We used the phylogenetic generalised least-squares method^72^,^73^ to explain the number of visible WNS lesions on a UV trans-illuminated wing based on *P. destructans* fungal load.

## Additional Information

**How to cite this article**: Zukal, J. *et al.* White-nose syndrome without borders: *Pseudogymnoascus destructans* infection tolerated in Europe and Palearctic Asia but not in North America. *Sci. Rep.* **6**, 19829; doi: 10.1038/srep19829 (2016).

## Supplementary Material

Supplemetary Tables S1-S3, Supplementary Figures S1, S2

## Figures and Tables

**Table 1:** Prevalence of white-nose syndrome and *Pseudogymnoascus destructans* infection in bat species from the Holarctic region.

Region	Bat species	Screened	WNS qPCR+		WNS UV+		WNS histo+	
		*n*	*n*	qPCR+ (%) ± s.e.	*n*	UV+ (%) ± s.e.	*n*	histo+ (%) ± s.e.
**Slovenia**	*Miniopterus schreibersii*	21	20	100 ± 7.1	21	47.62 ± 10.9	6	16.67 ± 20.05
	*Myotis emarginatus*	3	0	NA ± NA	3	100 ± 32.22	3	100 ± 32.22
	*Myotis myotis*	11	10	100 ± 13.21	11	63.64 ± 12.16	4	0 ± 26.89
	*Rhinolophus euryale*	14	14	64.29 ± 9.83	14	28.57 ± 9.83	1	100 ± 48.47
**Total**		**49**	**44**	**88.1** ± **4.88**	**49**	**59.96** ± **7**	**14**	**54.17** ± **13.32**
**Czech Republic**	*Barbastella barbastellus*	18	17	64.71 ± 11.59	18	50 ± 11.79	4	75 ± 26.89
	*Eptesicus nilssonii*	3	2	50 ± 39.61	2	0 ± 39.61	1	100 ± 48.47
	*Myotis alcathoe*	7	7	71.43 ± 17.76	7	14.29 ± 17.76	6	0 ± 20.05
	*Myotis bechsteinii*	23	23	86.96 ±6.23	23	39.13 ±10.18	9	44.44 ±14.45
	*Myotis brandtii*	17	16	68.75 ±8.71	17	23.53 ±8.24	1	100 ±48.47
	*Myotis dasycneme*	1	1	100 ±48.47	1	100 ±48.47	1	100 ±48.47
	*Myotis daubentonii*	33	30	86.67 ±4.85	31	51.61 ±8.98	14	35.71±12.81
	*Myotis emarginatus*	33	32	96.88 ±4.56	32	43.75 ±8.77	5	60 ±23
	*Myotis myotis*	156	150	99.33 ±1.01	108	96.3 ±1.4	61	73.77 ± 5.63
	*Myotis nattereri*	22	21	95.24 ±6.78	22	54.55 ±10.62	9	33.33 ±14.45
	*Plecotus auritus*	23	23	91.3 ± 6.23	22	68.18 ± 9.93	11	54.55 ± 12.16
	*Plecotus austriacus*	3	3	66.67 ± 32.22	3	33.33 ± 32.22	1	0 ± 48.47
	*Rhinolophus hipposideros*	35	29	79.31 ± 7.52	35	34.29 ± 8.02	8	50 ± 15.94
**Total**		**374**	**354**	**81.33** ± **2.07**	**321**	**46.84** ± **2.79**	**131**	**55.91** ± **4.34**
**Latvia**	*Eptesicus nilssonii*	4	4	25 ± 26.89	4	25 ± 26.89	1	0 ± 48.47
	*Myotis brandtii*	4	4	50 ± 26.89	4	50 ± 26.89	0	NA ± NA
	*Myotis dasycneme*	8	8	100 ± 15.94	8	100 ± 15.94	6	50 ± 20.05
	*Myotis daubentonii*	9	9	100 ± 14.45	9	88.89 ± 14.45	6	66.67 ± 20.05
**Total**		**25**	**25**	**68.75** ± **9.27**	**25**	**65.97** ± **9.48**	**13**	**38.89** ± **13.52**
**Russia**	*Eptesicus nilssonii*	5	5	100 ± 23	5	100 ± 23	2	100 ± 39.61
	*Myotis brandtii*	9	9	100 ±14.45	9	100 ± 14.45	2	0 ± 39.61
	*Myotis dasycneme*	17	17	100 ±8.24	17	100 ±8.24	12	75 ±11.27
	*Myotis daubentonii*	1	1	100 ± 48.47	1	100 ±48.47	0	NA ± NA
	*Plecotus auritus*	1	1	100 ± 48.47	1	100 ±48.47	1	100 ± 48.47
**Total**		**33**	**33**	**100** ± **4.43**	**33**	**100** ± **4.43**	**17**	**68.75** ± **11.24**
**USA**	*Myotis lucifugus*	10	0	NA±NA	10	100 ±13.21	10	100 ±13.21
**Total**		**10**	**0**	NA ± NA	**10**	**100** ± **13.21**	**10**	**100** ± **13.21**

Screened = number of bats captured and examined by PCR (to detect the pathogen), UV light trans-illumination or histopathology (to detect WNS lesions). + = percentage of positive bats from the number screened by the method. NA = not available. Samples subjected for histopathology examination were suspect lesions selected under field conditions based on UV trans-illumination and thus prevalence on histopathology is not based on a randomized sample. It rather reflects qualitative information that WNS was confirmed in the species with histopathology.
