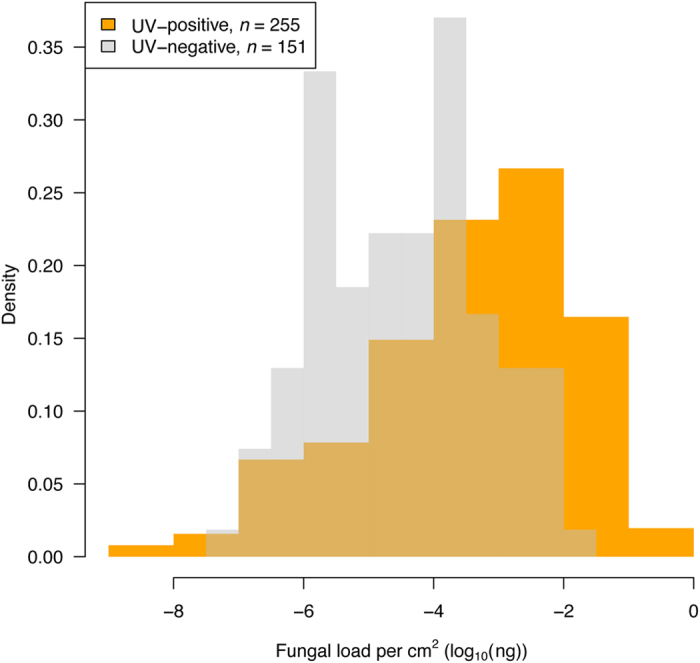# Corrigendum: White-nose syndrome without borders: *Pseudogymnoascus destructans* infection tolerated in Europe and Palearctic Asia but not in North America

**DOI:** 10.1038/srep26049

**Published:** 2016-05-20

**Authors:** Jan Zukal, Hana Bandouchova, Jiri Brichta, Adela Cmokova, Kamil S. Jaron, Miroslav Kolarik, Veronika Kovacova, Alena Kubátová, Alena Nováková, Oleg Orlov, Jiri Pikula, Primož Presetnik, Jurģis Šuba, Alexandra Zahradníková, Natália Martínková

Scientific Reports 6: Article number: 19829; 10.1038/srep19829 published online: 01292016; updated: 05202016.

This Article contains typographical errors.

In the Results section under subheading ‘Quantitative comparison of WNS on bats’,

“The fungal load on qPCR-positive bats ranged from 0.21 pg to 3.41 μg across the surface of the left wing (Supplementary Fig. S1, see Table 1 for sample sizes)”.

should read:

“The fungal load on qPCR-positive bats ranged from 0.21 fg to 3.41 ng across the surface of the left wing (Supplementary Fig. S1, see Table 1 for sample sizes)”.

“The fungal load from UV-negative individuals (median = 3.78 × 10^−5^ μg) overlapped that from UV-positive individuals (median = 7.46 × 10^−4^ μg; Fig. 8)”.

should read:

“The fungal load from UV-negative individuals (median = 3.78 × 10^−5^ ng) overlapped that from UV-positive individuals (median = 7.46 × 10^−4^ ng; Fig. 8)”.

In the upper panel of Figure 4, the y-axis ‘Fungal load per cm^2^ (log_10_(ng))’ was incorrectly given as ‘Fungal load per cm^2^ (log_10_(μg))’

In the upper panel of Figure 5, the y-axis ‘*P. destructans* load per cm^2^ (log_10_(ng))’ was incorrectly given as ‘*P. destructans* load per cm^2^ (log_10_(μg))’

In Figure 6, the x-axis ‘*P. destructans* load per cm^2^ (log_10_(ng))’ was incorrectly given as ‘*P. destructans* load per cm^2^ (log_10_(μg))’

In Figure 8, the x-axis ‘Fungal load per cm^2^ (log_10_(ng))’ was incorrectly given as ‘Fungal load per cm^2^ (log_10_(μg))’

In the legend of Figure 8,

“Bats were identified as positive (grey; *n* = 255) and negative (orange; *n* = 151) using UV trans-illumination”.

should read:

“Bats were identified as positive (orange; *n* = 255) and negative (grey; *n* = 151) using UV trans-illumination”.

The correct Figures 4, 5, 6 and 8 appear below as Figures 1, 2, 3 and 4 respectively.

## Figures and Tables

**Figure 1:**
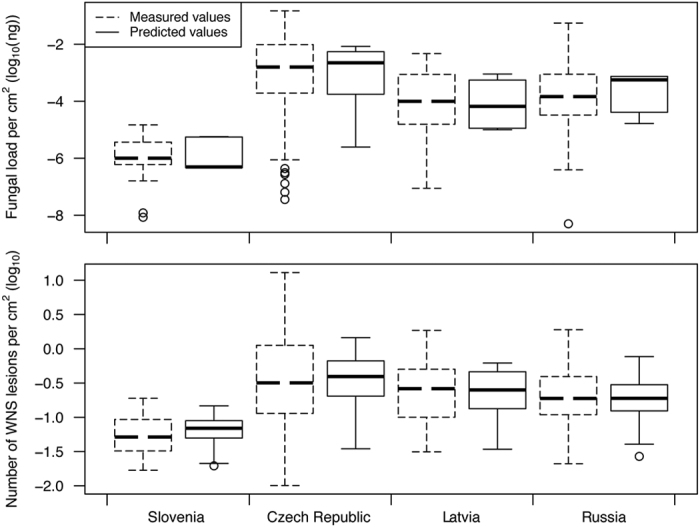


**Figure 2:**
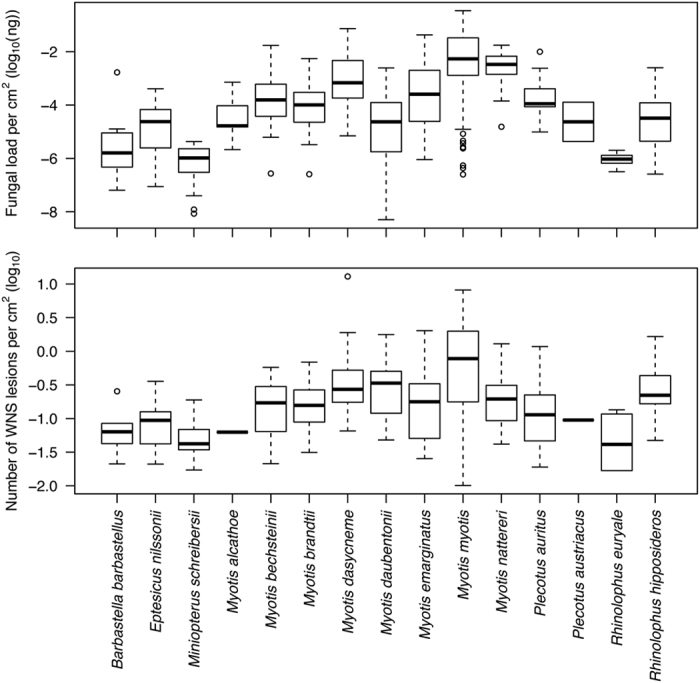


**Figure 3:**
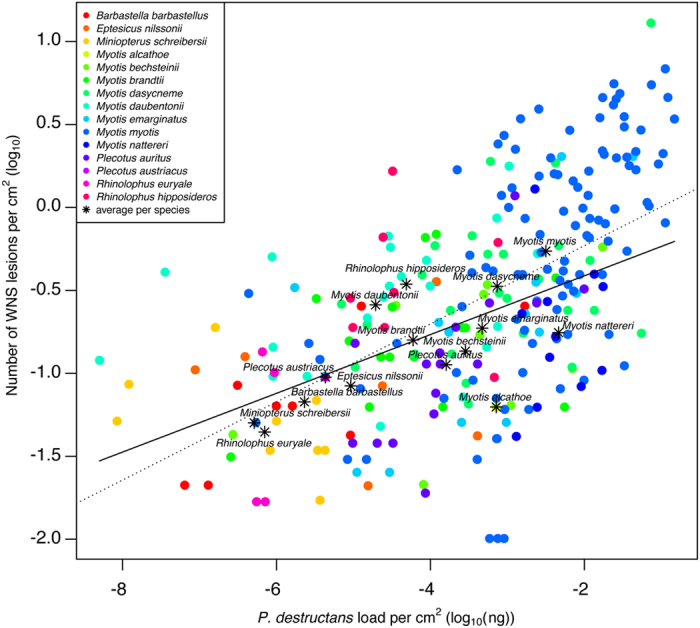


**Figure 4:**