# Supplementary material for: White-nose syndrome without borders: Pseudogymnoascus destructans infection tolerated in Europe and Palearctic Asia but not in North America
Source: Sci Rep. 2016 Jan 29;6:19829. doi: 10.1038/srep19829 (PMC4731777; doi:10.1038/srep19829)
Supplement: Supplemetary Tables S1-S3, Supplementary Figures S1, S2 [file srep19829-s1.pdf]

White-nose syndrome without borders: *Pseudogymnoascus destructans* infection tolerated in Europe and Palearctic Asia but not in North America

Jan Zukal, Hana Bandouchova, Jiri Brichta, Adela Cmokova, Kamil S. Jaron, Miroslav Kolarik, Veronika Kovacova, Alena Kubátová, Alena Nováková, Oleg Orlov, Jiri Pikula, Primož Presetnik, Jurģis Šuba, Alexandra Zahradníková, Jr. & Natália Martínková

Supplementary Table S1. Geographic coordinates of sites investigated for white-nose syndrome and *Pseudogymnoascus destructans* infection. Presence or absence of the disease was assessed using PCR (to detect the pathogen) and/or UV light trans-illumination or histopathology (to detect WNS lesions). Czech (CZ) bats were sampled between 2012 and 2014 and bats in Latvia (LV), Russia (RU) and Slovenia (SI) in 2014.

Country	Locality	Latitude	Longitude	PCR	UV	Histo	Screened
CZ	Sv. Kateřina Mine, Kašperské hory	49.27	13.1	+	+	+	12
CZ	Kristína Mine, Kašperské hory	49.13	13.58	+	+	-	10
CZ	Ledové sluje Cave, Vranov nad Dýjí	48.88	15.84	+	+	+	31
CZ	Nový Hrádek Castle	48.84	15.91	+	+	-	2
CZ	Býčí skála Cave, Moravský kras	49.31	16.69	+	-	-	1
CZ	Koňská jáma Cave, Moravský kras	49.36	16.71	+	-	-	3
CZ	Kateřinská Cave, Moravský kras	49.36	16.71	+	+	+	188
CZ	Stará Drátenická Cave, Moravský kras	49.29	16.73	-	-	-	1
CZ	Erichova Cave, Moravský kras	49.37	16.73	+	+	+	3
CZ	Nová Drátenická Cave, Moravský kras	49.29	16.73	+	+	-	2
CZ	Sloupsko-Šošůvské Caves, Moravský kras	49.41	16.74	+	+	+	4
CZ	Šimon-Juda Mine, Malá Morávka	50.05	17.3	+	+	+	117
SI	Puščava Church, Lovrenc na Pohorju	46.55	15.42	+	+	+	31
SI	Ajdovska jama Cave, Nemška vas, Krško	45.95	15.43	+	+	+	17
SI	Završe pri Grobelnem Church, Šentjur	46.23	15.44	-	+	-	1
LV	Bridge Strikupe (Vaidva) stream, Janmuiza	57.38	25.26	-	-	-	2
LV	Leila siksparnu Cave, Kazugrava	57.33	25.36	+	+	+	11
LV	Daugapils Fortress	55.89	26.49	+	+	+	12
RU	Slyudorudnik 2 Mine, Kyshtym, Chelyabinskaya oblast	55.68	60.35	+	+	+	12
RU	Smolinskaya Cave, Pokrovskoje, Sverdlovskaya oblast	56.43	61.61	+	+	+	21

Supplementary Table S2. Model selection with Akaike Information Criterion (AIC). Host species was treated as a random effect in all models.

**Model**	**AIC**	**ΔAIC**
Fungal load		
community + sampling date + latitude + longitude	769.54	
community + latitude+ longitude	774	4.46
community + latitude	790.93	21.39
community	796.76	27.22
community + sampling date	797.59	28.05
latitude+ longitude	821.78	52.24
sampling date + latitude + longitude	821.91	52.37
sampling date + latitude	822.44	52.9
sampling date	824.48	54.94
Number of WNS lesions		
fungal load + sampling date	381.52	
fungal load + sampling date + latitude	382.12	0.6
fungal load	382.84	1.32
fungal load + sampling date + latitude + longitude	383.8	2.28
fungal load + community	384.37	2.85
fungal load + latitude + longitude	384.51	2.99
fungal load + community + sampling date + latitude + longitude	384.94	3.42
community + sampling date + latitude + longitude	431.13	49.61

Supplementary Table S3. Primers used for PCR and sequencing.

Locus	Primer	Primer sequence 5' to 3'	Sequence length [bp]	Original reference for primer	Reference for PCR protocol
Nuclear ITS and partial LSU rDNA (*ITS*)	ITS1F	CTTGGTCATTTAGAG GAAGTAA	910	Gardes and Bruns (1993)	Minnis et al. (2013)
	ITS4	TCCTCCGCTTATTGAT ATGC		White et al. (1990)	
Translation elongation factor-1α (*TEF1α*)	EF1-983F	GCYCCYGGHCAYCGT GAYTTYAT	1038	Rehner and Buckley (2005)	Minnis et al. (2013)
	EF1-2218R	ATGACACCRACRGCR ACRGTYTG		Rehner and Buckley (2005)	
β-tubulin (*TUB2*)	T1	AACATGCGTGAGATT GTAAGT	400	Odonnell and Cigelnik (1997)	Pažoutová et al. (2015)
	T10	ACGATAGGTTCACCT CCAGAC		Odonnell and Cigelnik (1997)	
MAT1-1-1mating type (*MAT1-1-1*)	JP Pd MAT1-2-1 IntF	TCTCAGCTATAGTTT GCACCAA	523	Palmer et al. (2014)	Palmer et al. (2014)
	JP Pd MAT1-2-1 IntR	CATGCATGGGTATCA AAGCG		Palmer et al. (2014)	
MAT1-2-1mating type (*MAT1-1-1*)	JP GdMAT1-1 400F	AACAGGATCCGTTCA AAGCG	160	Palmer et al. (2014)	Palmer et al. (2014)
	JP Gd MAT1-1 400R	TGCCATGCCAAGAGG GTCC		Palmer et al. (2014)	
Calmodulin	CF1	GCCGACTCTTTGACY	789	Peterson et al. 2005	Nováková et al. (2014)

(*CAM*)		GARGAR	
	CF4	TTTYTGCATCATR AGYTGGAC	Peterson et al. 2005

References from Supplementary Table S3

Gardes M., Bruns T.D. (1993): ITS primers with enhanced specificity for basidiomycetes--application to the identification of mycorrhizae and rusts. Mol. Ecol. 2: 113-118.

Nováková A., Hubka V., Dudová Z., Matsuzawa T., Kubátová A., Yaguchi T. and Kolařík M. (2014): New species in *Aspergillus* section Fumigati from reclamation sites in Wyoming (U.S.A.) and revision of *A. viridinutans* complex. Fungal Diversity 64: 253-274.

Palmer J.M., Kubatova A., Novakova A., Minnis A.M., Kolarik M. and Lindner D.L. (2014): Molecular characterization of a heterothallic mating system in *Pseudogymnoascus destructans*, the fungus causing white-nose syndrome of bats. G3: Genes, Genomes, Genetics 4: 1755-1763.

Pažoutová S., Pešicová K., Chudíčková M., Šrůtka P. and Kolařík M. (2015): Delimitation of cryptic species inside *Clavicepspurpurea*. Fungal Biology 119: 7-26.

Peterson S.W., Vega F.E. and Posada F. (2005): *Penicillium coffeae*, a new endophytic species isolated from a coffee plant and its phylogenetic relationship to *P. fellutanum*, *P. thiersii* and *P. brocae* based on parsimony analysis of multilocus DNA sequences. Mycologia 97: 659-666.

O'donnell K., Cigelnik E. (1997): Two divergent intragenomicrDNA ITS2 types within a monophyletic lineage of the fungus *Fusarium* are nonorthologous. Mol. Phylogen. Evol. 7: 103-116.

Rehner S.A., Buckley E. (2005): A *Beauveria* phylogeny inferred from nuclear ITS and EF1-α sequences: Evidence for cryptic diversification and links to *Cordyceps* teleomorphs. Mycologia 97: 84-98.

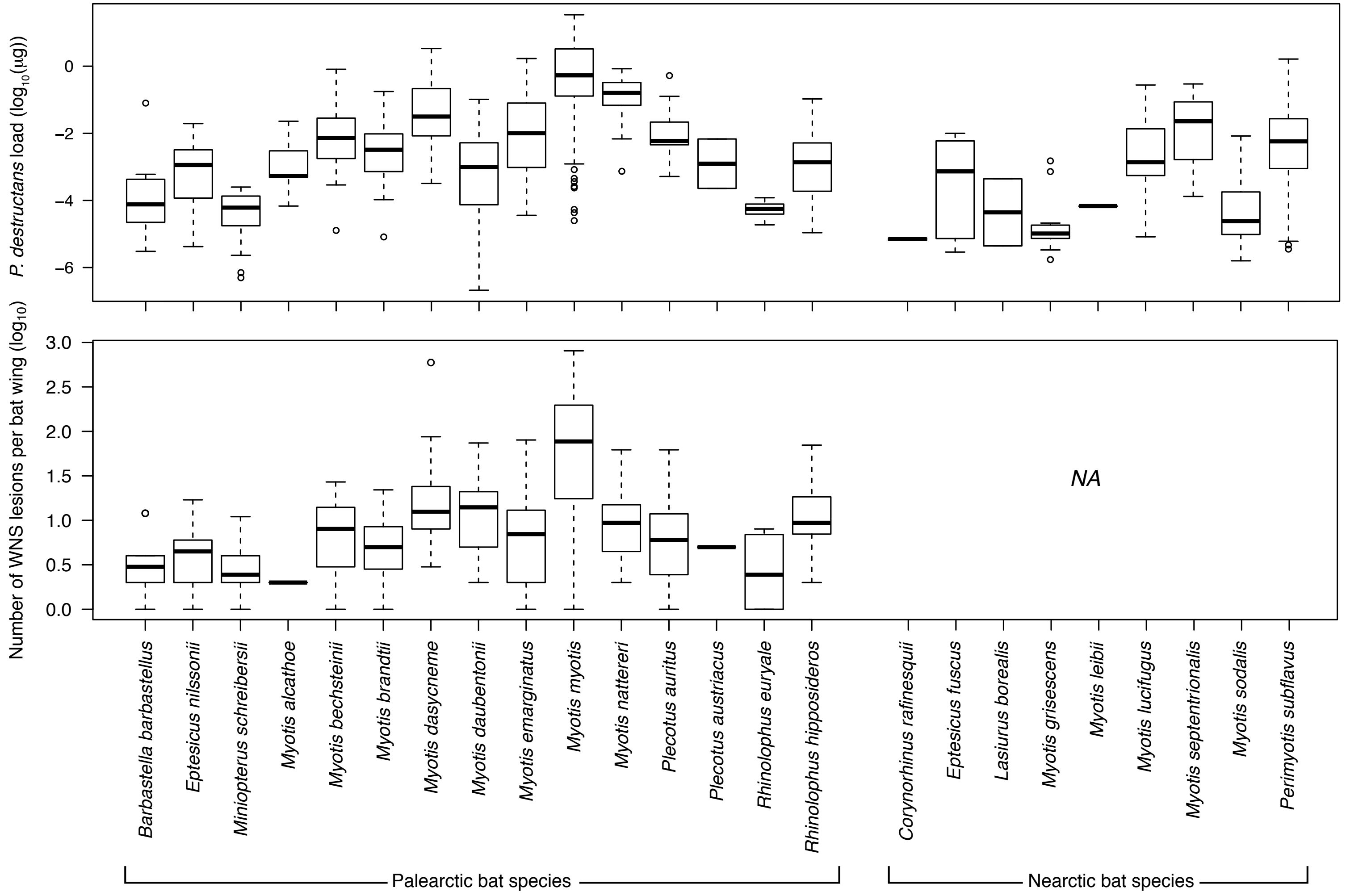

Supplementary Fig. S1. *Pseudogymnoascus destructans* fungal loads in bats from the Palearctic (Czech Republic, Latvia, Slovenia, Russia) and the Nearctic (USA). Data from Palearctic bats were detected using quantitative PCR across the dorsal side of the left wing and the number of WNS lesions using UV light trans-illumination. See main text for details on fungal load acquisition from Nearctic bats and the text and Table 1 for respective sample sizes. NA – not available.

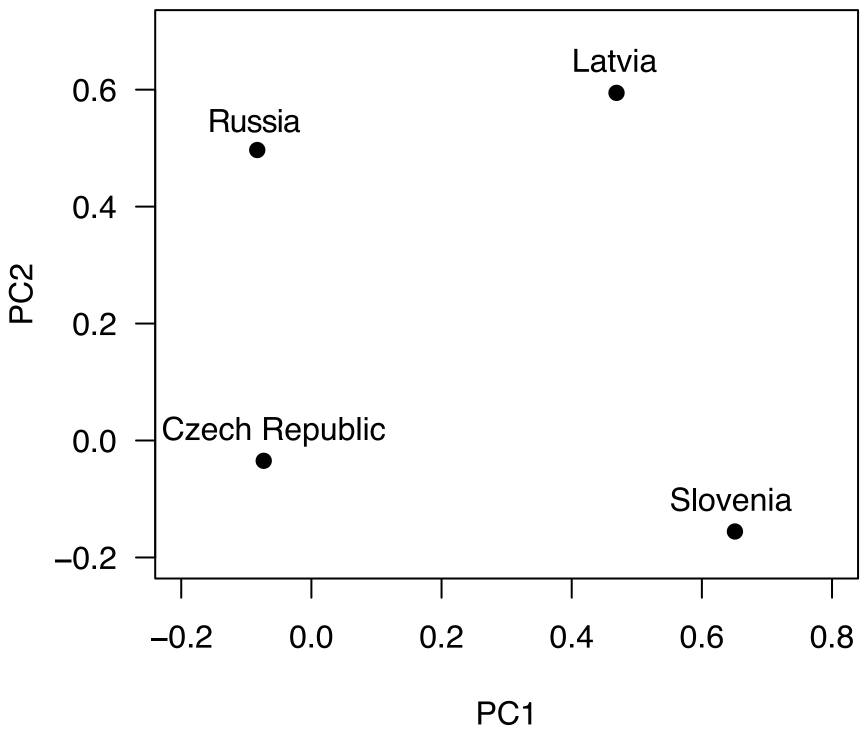

Supplementary Fig. S2. Relatedness of regional hibernating bat communities. Principal component analysis was calculated from mean phylogenetic and mean nearest taxon distances and UniFrac metric of specific bat communities at hibernacula at the given region.
